# Identification of Novel Potential Type 2 Diabetes Genes Mediating β-Cell Loss and Hyperglycemia Using Positional Cloning

**DOI:** 10.3389/fgene.2020.567191

**Published:** 2020-09-30

**Authors:** Heja Aga, Nicole Hallahan, Pascal Gottmann, Markus Jaehnert, Sophie Osburg, Gunnar Schulze, Anne Kamitz, Danny Arends, Gudrun Brockmann, Tanja Schallschmidt, Sandra Lebek, Alexandra Chadt, Hadi Al-Hasani, Hans-Georg Joost, Annette Schürmann, Heike Vogel

**Affiliations:** ^1^Department of Experimental Diabetology, German Institute of Human Nutrition Potsdam-Rehbrücke, Potsdam, Germany; ^2^German Center for Diabetes Research (DZD), München-Neuherberg, Germany; ^3^Animal Breeding Biology and Molecular Genetics, Albrecht Daniel Thaer-Institute for Agricultural and Horticultural Sciences, Humboldt University of Berlin, Berlin, Germany; ^4^German Diabetes Center (DDZ), Medical Faculty, Institute for Clinical Biochemistry and Pathobiochemistry, Heinrich Heine University Düsseldorf, Düsseldorf, Germany; ^5^Institute of Nutritional Science, University of Potsdam, Potsdam, Germany; ^6^Molecular and Clinical Life Science of Metabolic Diseases, University of Potsdam, Potsdam, Germany

**Keywords:** type 2 diabetes, β-cell loss, insulin, positional cloning, transcriptomics, haplotype

## Abstract

Type 2 diabetes (T2D) is a complex metabolic disease regulated by an interaction of genetic predisposition and environmental factors. To understand the genetic contribution in the development of diabetes, mice varying in their disease susceptibility were crossed with the obese and diabetes-prone New Zealand obese (NZO) mouse. Subsequent whole-genome sequence scans revealed one major quantitative trait loci (QTL), *Nidd/DBA* on chromosome 4, linked to elevated blood glucose and reduced plasma insulin and low levels of pancreatic insulin. Phenotypical characterization of congenic mice carrying 13.6 Mbp of the critical fragment of DBA mice displayed severe hyperglycemia and impaired glucose clearance at week 10, decreased glucose response in week 13, and loss of β-cells and pancreatic insulin in week 16. To identify the responsible gene variant(s), further congenic mice were generated and phenotyped, which resulted in a fragment of 3.3 Mbp that was sufficient to induce hyperglycemia. By combining transcriptome analysis and haplotype mapping, the number of putative responsible variant(s) was narrowed from initial 284 to 18 genes, including gene models and non-coding RNAs. Consideration of haplotype blocks reduced the number of candidate genes to four (*Kti12*, *Osbpl9*, *Ttc39a*, and *Calr4*) as potential T2D candidates as they display a differential expression in pancreatic islets and/or sequence variation. In conclusion, the integration of comparative analysis of multiple inbred populations such as haplotype mapping, transcriptomics, and sequence data substantially improved the mapping resolution of the diabetes QTL *Nidd/DBA*. Future studies are necessary to understand the exact role of the different candidates in β-cell function and their contribution in maintaining glycemic control.

## Introduction

Type 2 diabetes (T2D) arises from a complex interplay of multiple genetic and environmental factors that contribute to inadequate insulin secretion, insulin resistance, or both (American Diabetes Association, 2005; Tallapragada et al., 2015). A high percentage of obese people become diabetic, and the majority will at least experience insulin resistance and some degree of β-cell dysfunction (Kahn et al., 2006). Genetic predisposition accounts for differences in T2D susceptibility; so called “protective” gene variants mitigate the progression of the disease either through increased insulin sensitivity or secretion, while susceptibility variants can otherwise predispose an individual to β-cell failure and loss (McCarthy and Zeggini, 2009). Genome-wide association studies (GWAS) typically utilize genetic variants in the human population in the search for novel pathogenic genes. Despite the enormous numbers of human participants recruited for GWAS, diversity in the genome and environmental influences typically confound datasets, particularly for complex traits such as blood glucose or insulin concentrations. Thus, carefully controlled animal studies which allow for the precise manipulation of both environment and genetic variance remain a critical tool in T2D research.

In the past, individual crossbreeding approaches of inbred strains that vary in their predisposition to T2D have been useful for the identification of diabetes risk genes. However, with advances in bioinformatics and increasing capacity to deal with large and complex datasets, particularly involving the availability of genome-wide sequence coverage of common laboratory mouse strains, it has been advantageous to perform multiple crosses in parallel. We have recently carried out a collective diabetes cross (Vogel et al., 2018) using four lean inbred mouse strains (B6, DBA, C3H, and 129P2), each with varying susceptibility to T2D, to be individually crossbred with the obese and diabetes-prone New Zealand obese (NZO) mouse. This strategy addresses the current gaps in the knowledge of the genetic basis for T2D. It confers several advantages, including the ability to use haplotype maps for higher resolution positional cloning (Schwerbel et al., 2020).

The NZO mouse is an inbred strain that portrays a phenotype reminiscent of the human metabolic syndrome, displaying hyperphagia, severe obesity, insulin resistance, dyslipidemia, vascular disease, β-cell failure, and hyperglycemia (Kluge et al., 2012). With respect to islet dysfunction, NZO males display a more severe phenotype than females, and this gender difference is largely attributed to the protective effect of female sex hormones, in particular, estrogen (Vogel et al., 2013; Lubura et al., 2015). DBA mice, in contrast, are lean and normoglycemic. However, when obesity is induced with the introduction of either leptin or leptin receptor knockout mutations (lep-*ob* and lep-*db*, respectively), both male and female DBA mice develop a diabetes-like phenotype due to β-cell failure, indicating the presence of diabetes risk genes in the DBA genome (Leiter, 1981; Chua et al., 2002).

Here, we focus on the diabetes quantitative trait loci (QTL) *Nidd/DBA* on chromosome 4 which we identified as most prominent diabetes QTL in the NZOxDBA cross (Vogel et al., 2018). In the past, several QTL have been identified in the mid-region of mouse chromosome 4 in the proximity of the leptin receptor gene (*Lepr*) relating to hyperglycemia and hypoinsulinemia (Leiter et al., 1998; Plum et al., 2002; Scherneck et al., 2009; Davis et al., 2012; Kluge et al., 2012). In each case, the presence of a single allele from a lean, non-diabetic donor strain was able to produce a strong diabetogenic phenotype on an obese background. More specifically, the mice developed chronic hyperglycemia accompanied by pancreatic β-cell loss. Despite the fact that this trait has appeared repeatedly in crossbreeding experiments over the last 19 years, the responsible variant has yet to be identified. Variants in the gene *Zfp69* were previously hypothesized to be responsible for the diabetogenic effects of this locus (Scherneck et al., 2009). However, results obtained from subsequent characterization of *Zfp69*-tg mice suggested that *Zfp69* alone was not sufficient to trigger the hyperglycemic phenotype observed for the *Nidd/SJL* QTL (Scherneck et al., 2009; Chung et al., 2015). Here, we describe the QTL *Nidd/DBA*, a diabetogenic allele which was contributed by DBA and enhanced hyperglycemia and β-cell loss. In order to re-address the issue of which variants on chromosome 4 are responsible for this trait, *Nidd/DBA* recombinant congenic lines were generated on an NZO background. In the process of this investigation, cross-strain (NZO, B6, 129P2, DBA, and C3H) comparisons and bioinformatic approaches have been utilized in order to identify putative T2D genes.

## Materials and Methods

### Animals

Female NZO mice from our own colony (NZO/HIBomDife: German Institute of Human Nutrition, Nuthetal, Germany) and male DBA (DBA/2J: maintained in-house with breeders originating from Jackson Lab, Maine, United States) were crossed. Generation and phenotypical characterization of backcross mice were performed, as previously described (Vogel et al., 2018). Recombinant congenic mice were bred by repeated backcrossing of mice positive for the *Nidd/DBA* locus with NZO.

All congenic mice were maintained on chow diet (Ssniff, Soest, Germany; maintenance diet for rats and mice, product no. V153xR/M-H) containing 36% protein, 11% fat, and 53% carbohydrates. The animals were kept in accordance with EU Directive 2010/63/EU guidelines for the care and use of laboratory animals. All experiments were approved by the ethics committee of the State Agency of Environment, Health, and Consumer Protection (State of Brandenburg, Germany) under the permit numbers V3-2347-21-2012, 2347-10-2014, and 2347-13-2018.

### Genotyping of *Nidd/DBA* Mice by KASP and Microsatellite Markers

Genomic DNA was extracted from mouse tail-tips using the Invisorb Genomic DNA Kit II (STRATEC Molecular GmbH, Berlin, Germany), following the manufacturer’s instructions. Kompetitive allele-specific PCR (KASP) genotyping of N2 backcross mice was performed by LGC genomics (LGC group, Teddington, United Kingdom; Vogel et al., 2018). Recombinant congenic mice containing the *Nidd/DBA* locus were genotyped with KASP assays or by PCR with oligonucleotide primers obtained from Sigma (Sigma-Aldrich, Munich, Germany), and the microsatellite length was determined by non-denaturing polyacrylamide gel electrophoresis (Supplementary Table 1).

### Blood Glucose and Plasma Insulin

Random blood glucose was determined in the morning between 7 and 10 am with a Contour glucometer (Bayer, Leverkusen, Germany). Plasma insulin levels were determined by Mouse Ultrasensitive Insulin ELISA kit (Alpco, Salem, United States).

### Oral Glucose Tolerance Test

Oral glucose tolerance tests (oGTTs) were performed at 6 and 12 weeks of age. Animals were fasted for a period of 6 h and received 2 mg glucose (Glucosteril® 20%, Fresenius Kabi, Bad Homburg, Germany) per gram of body weight by oral gavage, subsequently. At the indicated time points, blood glucose and plasma insulin were obtained from the tail tip.

### Fasting-Refeeding Experiment

Fasting and refeeding experiments were performed at 13 weeks of age. After a 16 h overnight fasting period, blood glucose levels were determined and blood samples were collected. Subsequently, mice were refed for 2 h, and blood glucose levels and blood samples were collected.

### Endpoint Organ Collection

Mice were fasted 6 h before sacrifice. Blood was obtained by heart puncture with a 0.29 G needle attached to 0.5 M ethylenediaminetetraacetic acid (EDTA) coated syringes. Plasma was extracted by centrifugation (10,000 × *g*, 15 min, 4°C). Tissues were extracted and snap-frozen in liquid nitrogen. All tissue samples were stored at −80°C.

### Isolation of Pancreatic Islets

Islets were obtained from mouse pancreas following the protocol, as previously described (Kluth et al., 2015).

### Glucose-Stimulated Insulin Secretion in Primary Islets

Glucose-stimulated insulin secretion (GSIS) was performed, as described (Kluth et al., 2019). Briefly, 30 isolated and 24 h recovered islets were equilibrated in Krebs-Ringer buffer for 30 min under low glucose (2.8 mM) conditions and transferred into perifusion chambers (PERI5-115, Biorep Technologies Inc., Miami Lakes, FL, United States) with a continuous flow of 100 μl/min. GSIS was measured continuously under low (2.8 mM) and high glucose (20 mM) conditions and finally with low glucose for indicated periods. Fractions were collected in 2–3 min intervals. Insulin levels were measured using Mouse Ultrasensitive Insulin ELISA (Alpco) and normalized to residual insulin.

### Total Pancreatic Insulin

For detection of the pancreatic insulin content, whole pancreas was homogenized in ice-cold acidified ethanol (0.1 mol/L HCl in 70% ethanol) and incubated for 24 h at 4°C. After centrifugation (16,000 × *g*, 10 min), insulin was detected in 5 μl of the supernatant fraction using the Mouse High Range Insulin ELISA (Alpco) according to the manufacturer’s instructions.

### Immunohistochemistry and Immunofluorescence Staining

Embedded pancreatic sections were stained for insulin with the monoclonal mouse antibody K36AC10 (Sigma-Aldrich) by overnight incubation in a humid chamber at 4°C. For immunofluorescence staining, the secondary antibody 546 Alexa (Thermo Fisher Scientific Inc., Massachusetts, United States) and DAPI were applied at room temperature for 30 min. Next, biotinylated secondary antibody was applied and left at room temperature for 30 min. Subsequently, sections were treated with DAB substrate (DAB + Substrate Chromogen System, Agilent Technologies Inc.) for 2.5 min. Following staining of nuclei with hematoxylin and draining of the sections were performed in a Leica ST5020 Multistainer (Leica Biosystems, Wetzlar, Germany). For morphometric analysis, pancreatic sections were scanned with the MIRAX MIDI scanner (Carl Zeiss MicroImaging GmbH, Jena, Germany) and viewed with the MIRAX Viewer 1.12 software. Fluorescence images were examined with the confocal microscope Leica TCS SP8 (Leica Biosystems, Nussloch GmbH, Germany) and quantified by using the ImageJ software package [v1.52; Wayne Rasband (NIH)].

### Linkage Analysis

Genome-wide scan of N2 mice (NZOxDBA, *n* = 288 males/299 females) was performed, as previously described (Vogel et al., 2018). In brief, genetic map, genotyping errors, and linkage between individual traits and genotypes were assessed with the software package R/qtl (version 1.04-8) using the expectation maximization (EM)-algorithm and 1,000 permutations (Broman et al., 2003).

### Haplogroup Analysis

Haplogroup analysis was performed, as described (Schallschmidt et al., 2018). In brief, mouse single nucleotide polymorphism (SNPs) were used from the Wellcome Trust Sanger Institute Database.^1^ The chromosomal region was dissected into intervals of 250 kb to determine the frequency of polymorphic SNPs between the mouse strains. A window of 250 kb exceeding the threshold of 100 SNPs was defined as polymorphic according to the assumption (B6 = NZO ≠ DBA). For the total number of SNPs between parental strains, SNPs were compared to the C57BL/6J as reference.

### RNA Isolation

Isolation of total RNA from islets of Langerhans was performed with the Micro RNA Isolation RNAqueous® Kit (Life Technologies™, Darmstadt, Germany). Lysis of islet cells was initially carried out by a 10-s ultrasonic pulse (Branson Sonifier 450, G. Heinemann Ultraschall‐ und Labortechnik, Schwäbisch Gmünd, Germany). All further steps were carried out according to the manufacturer’s instructions.

### Genome-Wide mRNA Gene Expression

Whole-genome RNA-deep sequencing was performed by LGC Genomics (LGC Group). First, for data processing, adapters were trimmed and reads were filtered for quality using the wrapper Trim Galore and Cutadapt. Second, FastQC was utilized to check quality of samples, and finally, alignment of reads to reference genome was performed with HISAT2, and fragments per kilobase of transcript per million mapped reads (FPKM) values for transcripts have been determined by Cufflinks.

### Analysis of Putative Transcription Factor Binding Sites

For the identification of putative transcription factor binding sites (TFBSs) within the promotor region of differentially expressed genes, genetic variants (SNPs/Indels) between the parental strains NZO and DBA (Wellcome Trust Sanger Institute Database; REL-1505) were combined with position weight matrices (PWM) from the JASPAR database (Mathelier et al., 2014). TFBSs were predicted by R version 4.0 and the package TFBS tools (Tan and Lenhard, 2016).

### Statistics

Data, if not indicated otherwise, are presented as mean ± standard error of the mean (SEM). Statistical analyses were performed by either unpaired *t*-test, one-way ANOVA, or two-way ANOVA (with or without Bonferroni post-tests), as appropriate. GraphPad Prism 8 software (GraphPad, San Diego, United States) was utilized for simultaneous graph creation and statistical analysis. Significance levels were set for *p* < 0.05 (*), 0.01 (**), and 0.005 (***).

## Results

### *Nidd/DBA*, a Diabetes QTL, Contributed by DBA

**Figure 1:**
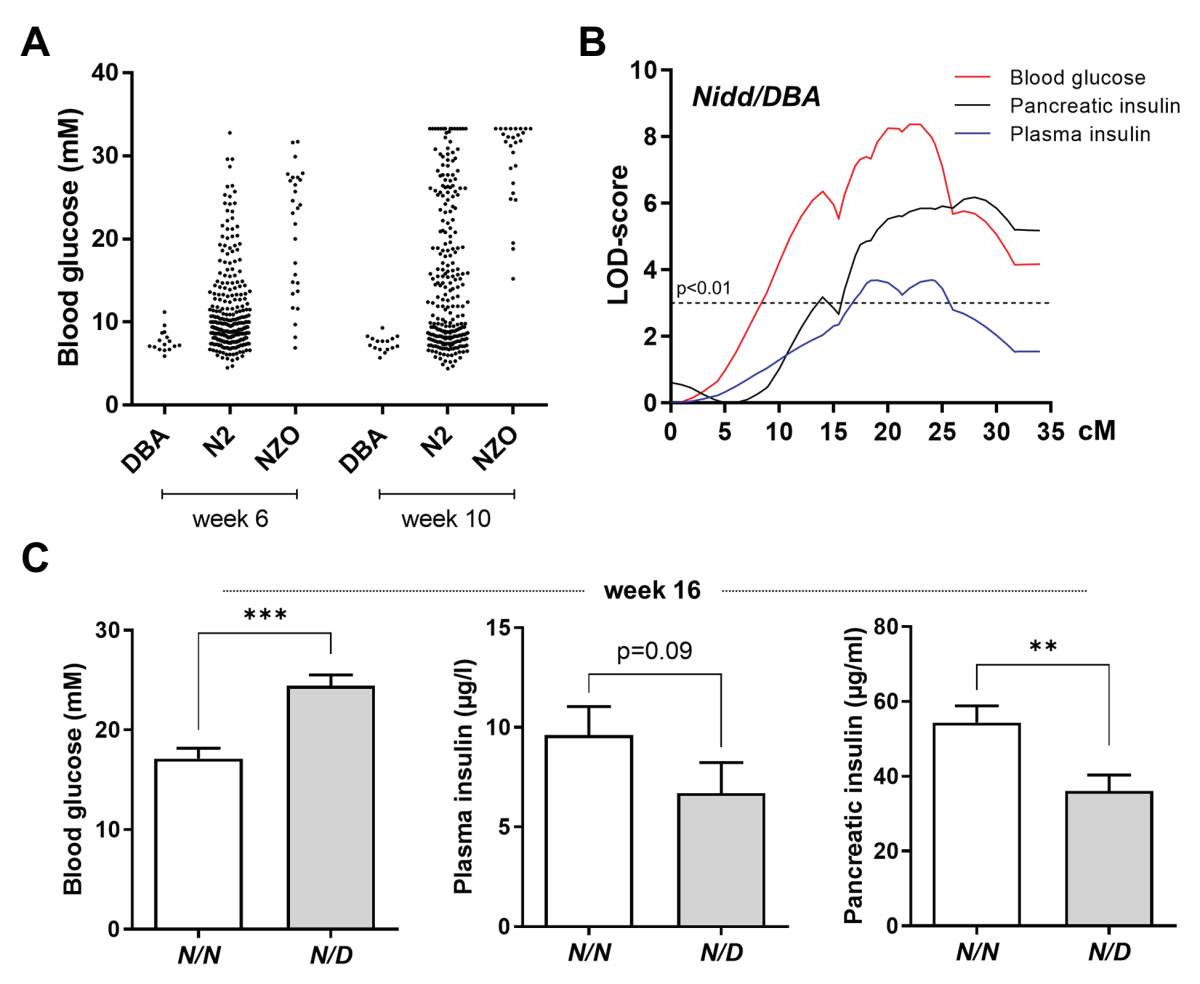
Localization of susceptibility loci on chromosome 4. **(A)** Distribution of blood glucose levels in the (NZOxDBA)N2 cohort at the age of 6 (DBA, *n* = 16; N2, *n* = 282; NZO, *n* = 28) and 10 (DBA, *n* = 16; N2, *n* = 219; NZO, *n* = 27) weeks in comparison to the parental NZO and DBA strains. **(B)** Genome-wide linkage analysis for the traits blood glucose, plasma insulin, and total pancreatic insulin revealed a single quantitative trait loci (QTL) on chromosome 4. The analysis was performed with data from male mice of the (NZOxDBA)N2 population at 16 weeks of age. The horizontal line indicates the threshold of significance (*p* < 0.01). **(C)** Effect sizes of traits associated with the QTL *Nidd/DBA* in the (NZOxDBA)N2 cohort. The critical region of *Nidd/DBA* was defined within a region of 46 Mbp on chromosome 4, defined by border SNP markers rs3692661 and rs3674038. Blood glucose (*N/N*, *n* = 103; *N/D*, *n* = 103), plasma insulin (*N/N*, *n* = 83; *N/D*, *n* = 52), and total pancreatic insulin (*N/N*, *n* = 80; *N/D*, *n* = 58) of homozygous (*N/N*) vs. heterozygous (*N/D*) N2 male mice for the critical region of the QTL. Data are presented as means ± SEM and were analyzed with Student’s *t*-test. ^**^p < 0.01; ^***^p < 0.005.

Male parental NZO and DBA mice were characterized on a 45% kcal high-fat diet (HFD) until the age of 16 weeks. The mice were phenotyped for metabolic traits relating to obesity and diabetes according to a standardized protocol that was established for the DZD collective diabetes cross (Vogel et al., 2018). NZO males, in contrast to DBA mice, developed overt diabetes, incidentally the average male blood glucose reading at the earliest time point measured (week 6) was >16.6 mM and all mice reached severe hyperglycemia (Figure 1A) combined with body weight loss by the endpoint of 16 weeks (data not shown). This heterogeneity observed between the two strains provided the basis for the subsequent investigation of QTL arising from the cross of both inbred strains. Since the trait blood glucose also scattered widely in the backcross population of NZO and DBA [(NZOxDBA)N2, Figure 1A], we assume that genetic variations are causal for the effect.

Indeed, in the (NZOxDBA)N2 population, a major QTL for elevated blood glucose, reduced plasma insulin concentrations, and low levels of total pancreatic insulin was localized to chromosome 4 with logarithm of the odds (LOD) scores of 8, 3.7, and 6, respectively (Figure 1B). This locus was designated *Nidd/DBA* (non-insulin dependent diabetes from DBA). As mice heterozygous for *NZO/DBA* (*N/D*) had an increased propensity toward hyperglycemia and low circulating and pancreatic insulin compared to homozygous *NZO/NZO* (*N/N*) allele carriers (Figure 1C), we concluded that the DBA genome contributed the diabetogenic gene.

### Characterization of the QTL *Nidd/DBA*

**Figure 2:**
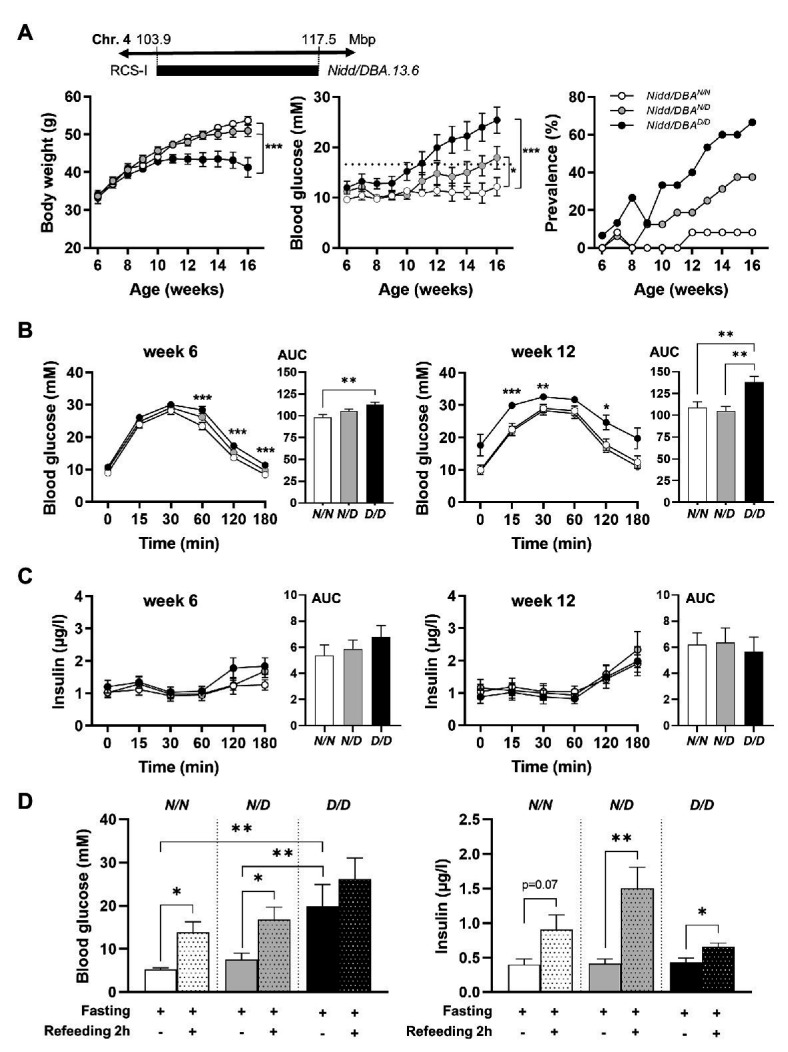
Diabetes traits of *Nidd/DBA* mice. **(A)** Male mice carrying 13.6 Mbp of chromosome 4 from NZO or DBA (*N/N*, *N/D*, and *D/D*) on the NZO background were characterized for the development of body weight (left panel), blood glucose (middle panel), and prevalence of diabetes (blood glucose >16.6 mM; right panel) on chow diet until 16 weeks of age. **(B)** Blood glucose and **(C)** insulin levels with corresponding area under the curve (AUC, right panels) during oral glucose tolerance test (oGTT) in male congenic mice at week 6 and week 12 (*N/N*, *n* = 12–19, *N/D*, *n* = 16–35, *D/D*, *n* = 12–20). **(D)** Blood glucose and insulin levels in 13-week-old *Nidd/DBA* mice after 16 h overnight fast and 2 h-refeeding (*N/N*, *n* = 6–8, *N/D*, *n* = 8–11, *D/D*, *n* = 4–5). Data are presented as means ± SEM and were analyzed by one-way ANOVA (AUC: **B**–**D**) or two-way ANOVA **(A–C)**. ^*^ *p* < 0.05; ^**^ *p* < 0.01; ^***^ *p* < 0.0005; *N/N*, *Nidd/DBA^N/N^* mice; *N/D*, *Nidd/DBA^N/D^* mice; *D/D*, *Nidd/DBA^D/D^* mice.

To analyze the phenotype conferred by the *Nidd/DBA* locus and to narrow down the critical region, recombinant congenic mice harboring one (RCS-I, *Nidd/DBA.13.6^N/D^*) or two diabetogenic (*Nidd/DBA.13.6^D/D^*) alleles from DBA on chromosome 4 (103.9–117.5 Mbp) on the NZO background were characterized and compared to homozygous NZO (*Nidd/DBA.13.6^N/N^*) controls (Figure 2A). From week 10 onward, blood glucose values were significantly higher in *Nidd/DBA.13.6^D/D^* mice compared to controls (Figure 2A). At the endpoint (16 weeks of age), approximately two-thirds (66.6%) of the *Nidd/DBA.13.6^D/D^* group was diabetic, as determined by blood glucose levels >16.6 mM and a stagnation or even drop in body weight, compared to 37.5% diabetic *Nidd/DBA.13.6^N/D^* and 8.3% *Nidd/DBA.13.6^N/N^* mice. Likewise, we observed an impaired glucose tolerance during oGTTs in homozygous *Nidd/DBA* allele carriers compared to controls. This effect worsened progressively from week 6 to week 12 (Figure 2B). However, the corresponding plasma insulin levels during the oral glucose tests were similar between the different groups (Figure 2C).

**Figure 3:**
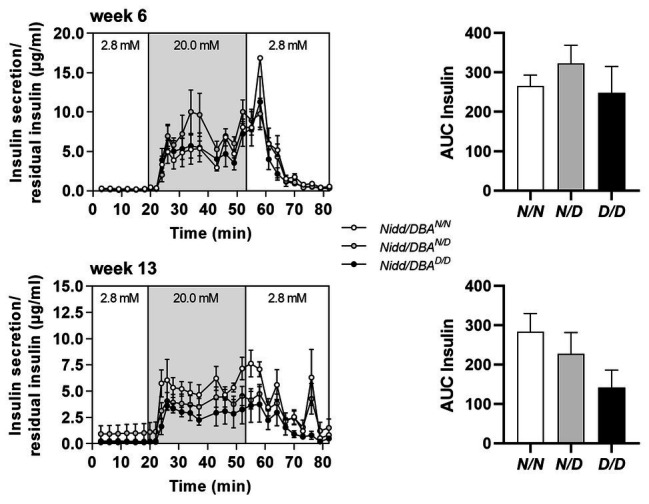
Insulin secretion in *Nidd/DBA* islets *ex vivo*. Glucose-stimulated insulin secretion (GSIS) in primary islets of 6 (*n* = 3) and 13 weeks old homozygous NZO (*N/N*, *n* = 4), heterozygous (*N/D*, *n* = 6), or homozygous DBA (*D/D*, *n* = 3) allele carriers for the *Nidd/DBA* locus (RCS-III, *Nidd/DBA.6.3*) with corresponding AUC (right panel). Data are presented as means ± SEM and were analyzed by one-way ANOVA.

To further estimate glucose homeostasis and insulin sensitivity, we performed fasting-refeeding experiments in congenic mice at the age of 13 weeks. In the fasting state (16 h fasting), homozygous DBA mice had already higher blood glucose levels than heterozygous and homozygous NZO mice, whereas circulating insulin levels were identical between the different groups (Figure 2D). After 2 h refeeding, the homozygous *Nidd/DBA.13.6^N/N^* and heterozygous *Nidd/DBA.13.6^N/D^* mice showed significantly higher blood glucose and insulin levels compared to the corresponding fasting state. Although the homozygous *Nidd/DBA.13.6^D/D^* mice were hyperglycemic already in the fasting state, the mice were still able to secrete significantly more insulin under re-fed conditions (Figure 2D). Moreover, in isolated islets of 13-week-old *Nidd/DBA.13.6^D/D^* and *Nidd/DBA.13.6^N/N^* mice, we tested the capacity for GSIS. During the challenge, *Nidd/DBA.13.6^D/D^* and *Nidd/DBA.13.6^N/N^* islets displayed equivalent basal (2.8 mM media glucose concentration) insulin secretion capacity. In response to 20 mM glucose, *Nidd/DBA.13.6^D/D^* islets showed a tendency toward a reduced insulin secretion without reaching significance. No differences in the insulin secretion capacity were obtained in islets of 6 weeks old mice (Figure 3).

### *Nidd/DBA* Mice Exhibited Marked β-Cell Loss and Islet Destruction

**Figure 4:**
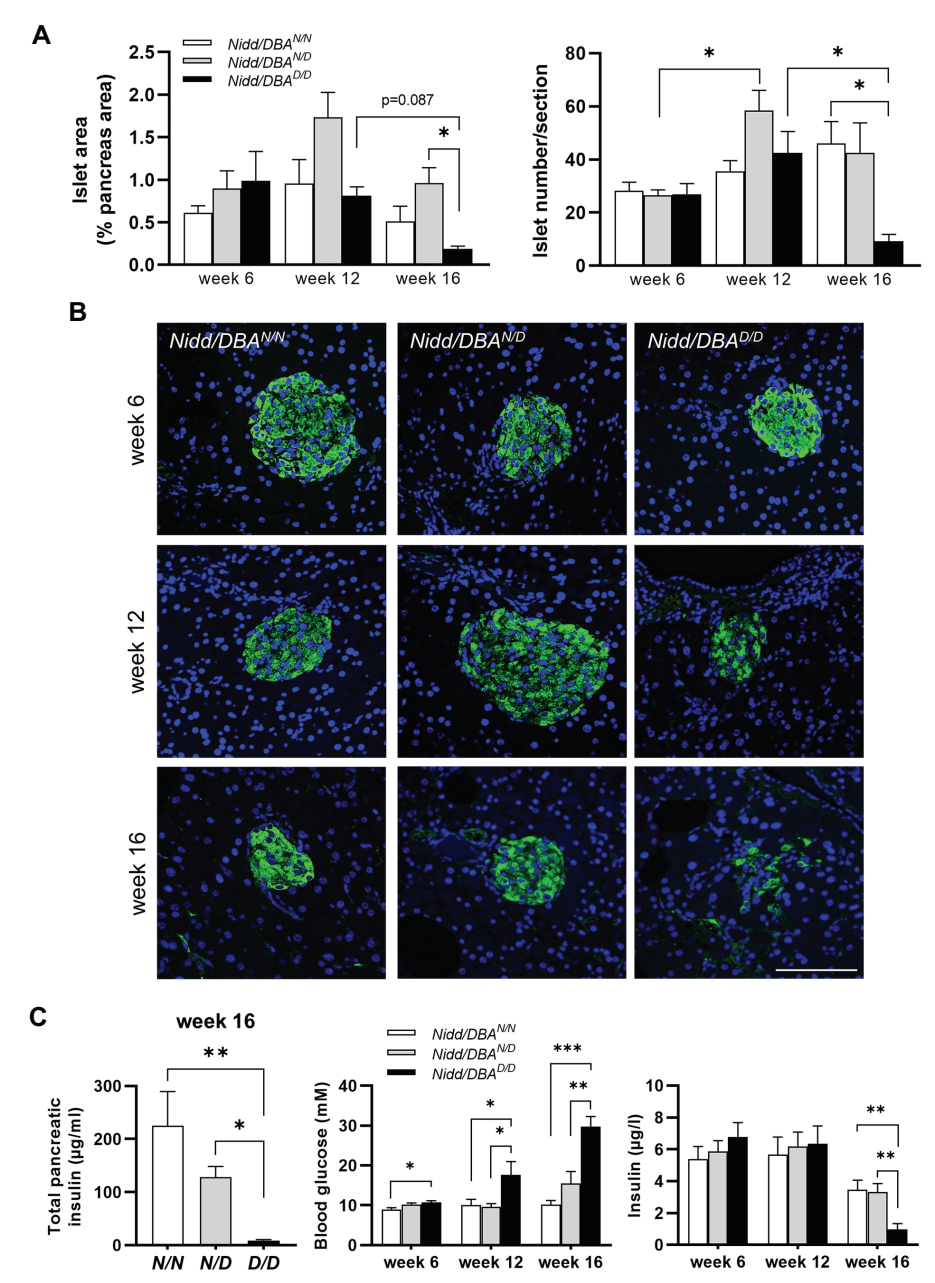
*Nidd/DBA* mice exhibited marked β-cell loss and islet destruction. **(A)** Quantification of islet area (left panel) and islet number/section (right panel) at the indicated time points in male *Nidd/DBA^N/N^* (*N/N*), *Nidd/DBA^N/D^* (*N/D*), and *Nidd/DBA^D/D^* (*D/D*) mice (*n* = 3–8 animals/genotype). **(B)** Representative images of pancreatic sections of male *Nidd/DBA* (RCS-I) mice stained for insulin (green). Nuclei were stained with DAPI (blue); Scale bar: 100 μm. **(C)** Pancreatic insulin content in congenic mice (RCS-I) at the age of 16 weeks (*n* = 5–7) and 6 h fasting blood glucose and insulin values in RCS-I mice at different time points (*N/N*, *n* = 5–19, *N/D*, *n* = 6–35, *D/D*, *n* = 10–20). Data are presented as means ± SEM and were analyzed by one-way ANOVA. ^*^p < 0.05; ^**^p < 0.01; ^***^p < 0.0005.

As we observed higher blood glucose concentrations in *Nidd/DBA.13.6^D/D^* mice, we isolated the pancreas of congenic mice at different time points to monitor the islet morphology. As shown in Figure 4A and Supplementary Figure S1, at 16 weeks of age, the islet area, as well as the number of islets, was significantly reduced in homozygous *Nidd/DBA.13.6^D/D^* mice compared to heterozygous allele carriers and tendentially also to homozygous controls (*Nidd/DBA.13.6^N/N^*), assuming in combination with the hyperglycemic phenotype that *Nidd/DBA.13.6^D/D^* mice exhibited a marked loss in pancreatic β-cells. Immunofluorescent staining of pancreatic islets with an insulin specific antibody additionally highlighted the loss of β-cells in *Nidd/DBA.13.6^D/D^* mice (Figure 4B) with a gradual loss from week 6 to week 16. In order to validate the data obtained by immunohistochemistry, we determined the insulin content of total pancreas by immunoassay of acidic ethanol extracts (Figure 4C). A significant decrease of the insulin content in *Nidd/DBA.13.6^D/D^* mice was detected at the age of 16 weeks. Thus, a gradual decrease in total pancreatic insulin appeared to accompany the increase in fasting plasma glucose levels combined with a fall in fasting plasma insulin levels (Figure 4C).

### Fine Mapping of the Critical Diabetogenic Interval of *Nidd/DBA*

**Figure 5:**
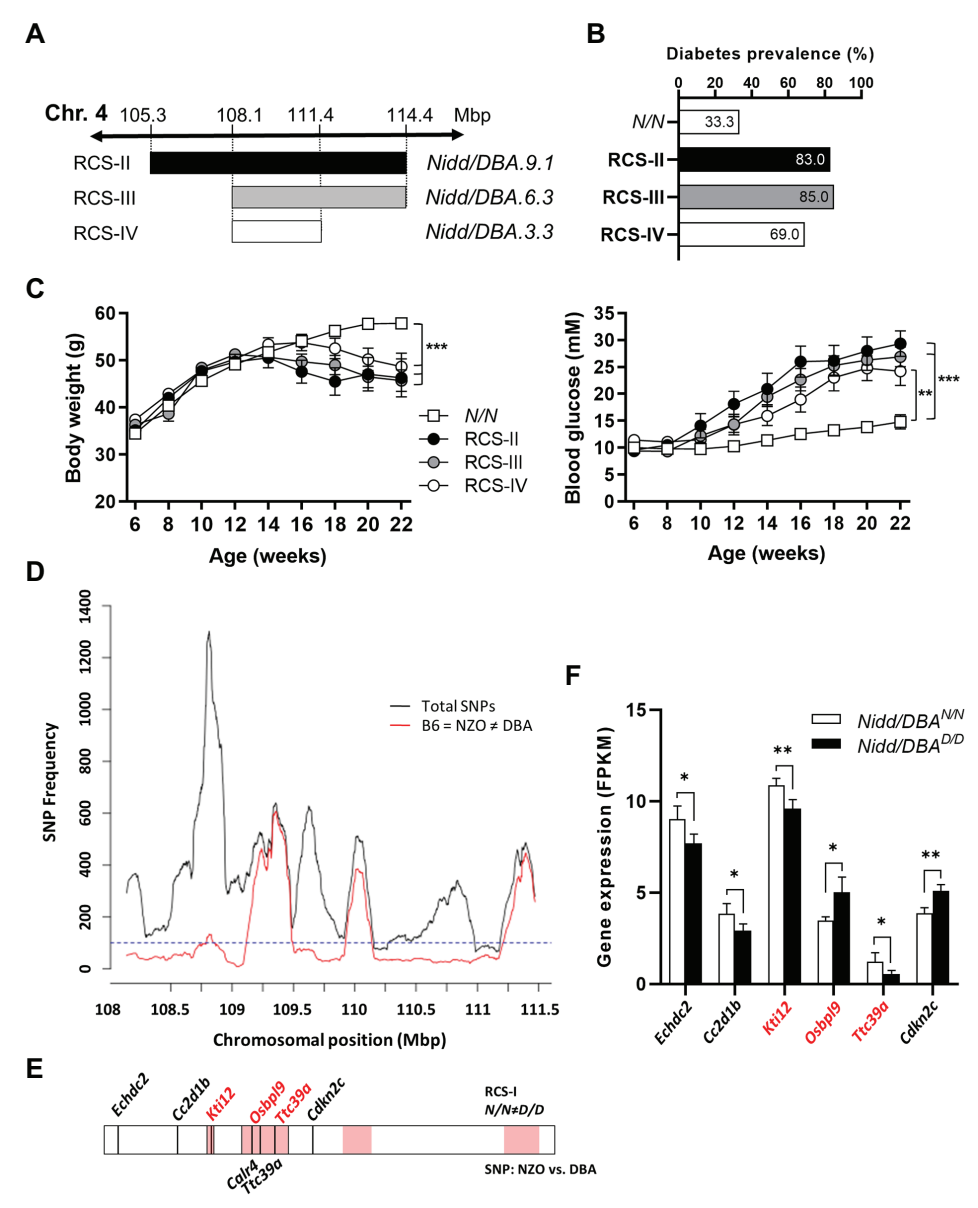
Combined approach of haplotype and gene expression analysis in islets for the identification of DBA-specific gene variants within the *Nidd/DBA* locus. **(A)** Generation of congenic mice carrying different fragments of the QTL *Nidd/DBA*. **(B)** Diabetes prevalence (blood glucose >16.6 mM, week 16), **(C)** body weight, and blood glucose development in congenic mice carrying one heterozygous DBA allele for the *Nidd/DBA* locus compared to homozygous NZO controls. **(D)** Haplotype map of single nucleotide polymorphisms (SNPs) within the critical region of *Nidd/DBA* (108.8–111.4 Mbp). Black line: total number of SNPs (all SNPs annotated for the B6 reference genome with calls for NZO and DBA). Red line: SNPs according to B6 = NZO ≠ DBA. **(E)** QTL was dissected into windows of 250 kb. Red boxes indicate 100 SNPs per window polymorphic according to B6 = NZO ≠ DBA. Location of genes within the critical *Nidd/DBA* interval with a differential expression in pancreatic islets of 6 weeks old congenic mice (*N/N* ≠ *D/D*) and position of genes with non-synonymous coding variants between the parental strains NZO and DBA (REL-1505-GRCm38). **(F)** Genes within the critical *Nidd/DBA* interval displaying a differential expression in pancreatic islets of congenic mice (RCS-I, *Nidd/DBA.13.6*). Expression was analyzed *via* RNA-sequencing (*n* = 4). Genes with differential expression in pancreatic islets and location within a polymorphic *Nidd/DBA* haplotype block are indicated in red. Data are presented as means ± SEM and were analyzed with Student’s *t*-test **(F)** or one-way ANOVA **(C)**. ^*^p < 0.05; ^**^p < 0.01; ^***^p < 0.001.

The region spanning 13.6 Mbp of the *Nidd/DBA* harbors 284 genes including 139 annotated genes, 78 gene models, and 67 non-coding RNAs (Yates et al., 2020). To identify the responsible gene variant(s), additional recombinant congenic lines, each carrying a distinct region of the initial *Nidd/DBA* locus, were generated by further backcrossing to NZO (Figure 5A). The characterization of these mice indicated that more than two-thirds of the heterozygous allele carriers of each line developed a diabetic phenotype at the endpoint of 16 weeks, compared to 33.3% diabetic *Nidd/DBA^N/N^* mice (Figure 5B). Heterozygous mice of each line developed hyperglycemia compared to normoglycemic controls, and the body weight already started to drop (Figure 5C). Thus, a 3.3 Mbp fragment with 61 genes including 28 annotated genes, 18 gene models, and 15 non-coding RNAs (Yates et al., 2020), comprising the diabetogenic allele, was defined to be located between 108.1 Mbp (rs233757491) and 111.4 Mbp (rs32727600).

Within the collective diabetes cross project, a backcross population of the NZO and B6 strain was conducted, where no QTL on chromosome 4 with similarities to the *Nidd/DBA* locus was defined. Thus, the causal gene variant(s) may be common between the NZO and B6 strain but different to DBA. According to this hypothesis, a haplotype map of the putative critical region on chromosome 4 was created based on NZO/HiltJ, DBA/2J, and C57BL/6J (B6) SNP information (Keane et al., 2011; Yalcin et al., 2011). A graphical representation of the polymorphic regions found in the *Nidd/DBA* locus is shown in Figure 5D, where several clusters of variants can be identified across the entire region. In total, 18 genes (11 annotated genes, one gene model, three processed pseudo genes, and three non-coding RNAs) were found to be unique for the DBA strain but different to NZO and B6.

Moreover, gene expression profiles of pancreatic islets were generated from congenic mice carrying the initial 13.6 Mbp fragment (RCS-I) of the *Nidd/DBA* locus. Six genes within the critical region were differentially expressed in pancreatic islets of homozygous *Nidd/DBA^D/D^* mice compared to homozygous *Nidd/DBA^N/N^* controls (Figure 5F), whereby only *Kti12*, *Osbpl9*, and *Ttc39a* are also located in a polymorphic haplotype block (Figure 5E). As the complete NZO and DBA genomic sequence is publicly available, we also screened for sequence variations in genes located within the polymorphic *Nidd/DBA* haplotype block. In total, two non-synonymous SNPs (Table 1) in association with *Ttc39a* [rs28150999; missense variant (P169L)] and *Calr4* (rs219833914; frameshift variant) were found to be common between NZO and B6 but different to DBA (Table 1; Figure 5E).

**Table 1:** Summary of genetic variants in the critical region of *Nidd/DBA*, common between the New Zealand obese (NZO) and B6 strain but different to DBA (Keane et al., 2011; Yalcin et al., 2011).

Variant type	Number of SNPs	Number of InDels	Number of genes
Intron	1,139	489	29
Upstream	326	114	30
Missense	1	-	1
Splice region	-	2	2
Frameshift	-	1	1
Stop	-	-	-
Intergenic	433	152	0

The region defining upstream variants was set by 2 kbp upstream of the transcription start site.

Next, we aimed to investigate the underlying genetic cause of the differential expression of *Ttc39a*, *Osbpl9*, and *Kti12*. An *in silico* approach using a PWM based TFBS prediction revealed strong alterations for eight transcription factors (TFs) in a region 2 kbp upstream of *Ttc39a* (Supplementary Table 2). These variants lead to an altered core region in the DBA sequence, and it is possible that these sequence alterations and a likely difference in TF binding are causal for the low *Ttc39a* expression in DBA allele carriers.

### Expression of Human Orthologues of *Nidd/DBA* Candidates in Patients With Type 2 Diabetes

**Figure 6:**
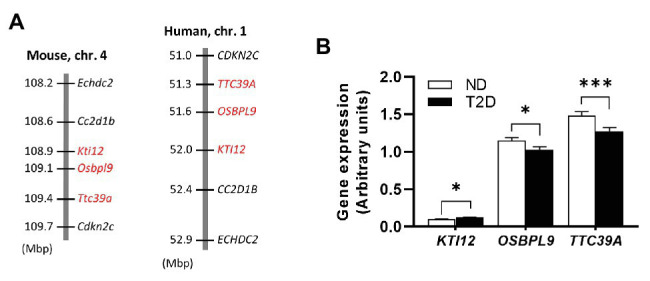
Expression of *Nidd/DBA* candidate genes in human pancreatic islets. **(A)** Cluster of the critical *Nidd/DBA* region on mouse chromosome 4 and human chromosome 1. **(B)** Expression of candidate genes in pancreatic islets of T2D (*n* = 26) and normoglycemic controls (*n* = 51; Fadista et al., 2014). Data are presented as means ± SEM and were analyzed with Student’s *t*-test. ^*^p < 0.05; ^***^p < 0.0005.

The *Nidd/DBA* locus on mouse chromosome 4 is syntenic to the human genomic segment on chromosome 1 (Figure 6A). To clarify the importance of the genes with differential expression in mouse islets in the pathogenesis of T2D, we used transcriptomic data from human pancreatic islets of deceased donors with and without T2D (Fadista et al., 2014). As shown in Figure 6B, all three genes displayed a significantly different expression in islets of patients with and without T2D, whereby only the effect for *TTC39A* with a reduced expression in T2D patients was comparable with the data obtained in pancreatic islets of mice carrying the diabetogenic *Nidd/DBA* allele (Figure 5F).

## Discussion

The present data define a critical interval of the QTL *Nidd/DBA* on chromosome 4, a diabetogenic allele which was introduced by DBA. The QTL is characterized by hyperglycemia starting at 10 weeks of age, accompanied by hypoinsulinemia and a loss of body weight and total pancreatic insulin levels at the endpoint of the study at 16 weeks. In order to identify the responsible variant(s) on chromosome 4, *Nidd/DBA* recombinant congenic lines were generated and combined with transcriptomics and different bioinformatic tools to finally define a critical QTL segment of 3.3 Mbp which contains four candidate genes for the observed diabetic phenotype.

The diabetogenic allele of *Nidd/DBA* mice induces severe hyperglycemia and impaired glucose clearance starting at 10 weeks of age and decreased glucose response in week 13. In week 6, *Nidd/DBA^D/D^* islets displayed equivalent basal as well as GSIS, whereas in week 13, carriers of the diabetogenic allele showed a tendency of an impaired insulin secretion capacity. Also, the unchanged insulin levels during the glucose tolerance tests which showed an impaired glucose clearance indicate that *Nidd/DBA^D/D^* mice exhibit a restricted insulin secretion. Histology of non-diabetic *Nidd/DBA^N/N^* and *Nidd/DBA^D/D^* mice at the age of 6 weeks did not indicate any early differences in islet morphology, whereas later immunostaining of pancreatic sections of diabetic *Nidd/DBA* mice revealed a loss of β-cells and reduced islet size to be a central element in the pathology of *Nidd/DBA* induced T2D. The time course data indicate that, presumably, due to a moderate and early impairment of insulin secretion, a fast β-cell loss occurs within a period of 3–4 weeks.

Many mouse-cross populations have been analyzed worldwide, but despite the fact that diabetes-related QTL on chromosome 4 have appeared several times in both DBA and NZO outcrosses (BKS-db/−xNZO: Togawa et al., 2006; NONxNZO: Leiter et al., 1998; NZOxSJL: Plum et al., 2002), the responsible variant has yet to be elucidated. The gene *Zfp69*, uniquely expressed in SJL and repressed in NZO due to the presence of a retrotransposon, is located on chromosome 4 at approximately 121 Mbp and was previously investigated as a T2D candidate, but does not account for islet dysfunction and β-cell loss (Scherneck et al., 2009; Chung et al., 2015). DBA mice carry an intact copy of *Zfp69*, however, characterization of recombinant congenic mice excluded the possibility that *Zfp69* is responsible for β-cell failure observed in *Nidd/DBA* mice, as *Zfp69* is located outside of the smallest critical *Nidd/DBA* fragment (108.1–111.4 Mbp) inducing hyperglycemia. In addition to SJL, QTL for elevated blood glucose were identified on chromosome 4 from parallel NZO crosses with C3H and 129P2 (Schallschmidt et al., 2018). The QTL derived from SJL, 129P2, and C3H were characterized not only by hyperglycemia but also by a lack of insulin, indicating that β-cell failure is underlying the diabetogenic effect of the QTL. However, it is not entirely clear whether the DBA, C3H, 129P2, and SJL strains harbor the same pathological variant on chromosome 4. Therefore, for haplotype mapping, we just included the sequence variants in the critical region of the *Nidd/DBA* between DBA and NZO as well as the B6 strain, as no QTL for hyperglycemia was detected on chromosome 4 in the (NZOxB6)N2 cross.

Of the 18 genes within the haplotype block that carry common variants between these strains, three genes located between 108.1 and 111.4 Mbp are differently expressed in islets of homozygous DBA compared to homozygous NZO mice. One of these genes, *Ttc39a* [tetratricopeptide repeat (TPR) domain] also carries a missense variant in the DBA genome. *Ttc39a* belongs to the structural family of TPR domain proteins, which comprises about 100 members characterized by repeats of a 34-amino-acid consensus (Dmitriev et al., 2009). The TPR-containing (TTC) proteins are involved in many important biological processes, such as intracellular transport, vesicle fusion, protein folding, cell cycle, and transcriptional regulation (Zeytuni and Zarivach, 2012; Xu et al., 2015). For *Ttc4*, it has been demonstrated that it is involved in the intraflagellar transport (IFT), a trafficking machinery essential for cilia formation and function (Hao and Scholey, 2009). Defects in cilia assembly and function result in severe disorders called ciliopathies (Xu et al., 2015). A subset of ciliopathies including Bardet-Biedl-Syndrome (BBS) and Alström Syndrome (ALMS) shows metabolic abnormalities including truncal obesity and higher susceptibility to diabetes (Volta and Gerdes, 2017). Cilia are present, e.g., in endocrine β-cells, and it has been reported that cilia play a role in insulin signaling, insulin secretion, and β-cell function (Gerdes et al., 2014). We recently reported that cilia-gene regulation has a strong impact on the presence and dynamics of cilia in islets which, in turn, determines diabetes susceptibility (Kluth et al., 2019). However, as there are multiple transcript variants described for *Ttc39a*, which encode distinct isoforms, the specific role of the differently expressed isoform in the pathogenesis of diabetes in mice has to be clarified. The second gene with a differential expression is *Kti12* (*KTI12 homolog*, *chromatin associated*). *Kti12* is known to be a vastly conserved ATPase and essential for transfer RNA (tRNA)-modification activity of the Elongator complex (Krutyhołowa et al., 2020). So far, *Kti12* was mainly described in plant cell biology, where it plays a role in the transcription by interacting with RNA polymerase II (Nelissen et al., 2003). Attempts to generate *Kti12*-KO models in mice resulted either in embryonic lethality (prior to organogenesis) or pre-weaning lethality (IMPC^2^). The gene *Osbpl9* (*oxysterol binding protein-like 9*) belongs to the oxysterol-binding protein family, which has been implicated in the function of, e.g., endoplasmic reticulum (ER) junctions, non-vesicular transport of lipids, integration of sterol and sphingomyelin metabolism, sterol transport, regulation of neutral lipid metabolism, and regulation of signaling cascades (Olkkonen et al., 2006). Familial loss-of-function variants of OSBPL1 in humans predisposed to impaired reverse cholesterol transport and low plasma high-density lipoprotein (HDL) levels (Motazacker et al., 2016). Studies on OSBPL10 suggested a suppressive function on hepatic lipogenesis and very low density lipoprotein (VLDL) production (Perttilä et al., 2009). Finally, the gene *Calr4* (*Calreticulin 4*) located within the critical *Nidd/DBA* segment carries a frameshift mutation within the first exon in the DBA strain. *Calr4* alongside with *Crt* (*calreticulin*), *Cnx* (*calnexin*), and *Clgn* (*calmegin*) belongs to the calreticulin protein family that is known to be involved in calcium binding. Genes of the calreticulin family encode for proteins located in the endoplasmic reticulum (ER; Michalak et al., 1992; Bergeron et al., 1994; Watanabe et al., 1994). *Crt* promotes folding and quality control of the ER by interactions with *Cnx* (Calreticulin/calnexin cycle; Raghubir et al., 2011). However, due to the fact that *Calr4* was not detected in islets by RNAseq, we concluded that its variant is not involved in the impaired β-cell function induced by *Nidd/DBA*. In addition, *Calr4* appears to only be expressed during embryonic development in mice, it is poorly conserved and only encoded as a pseudogene in humans.

In summary, the positional cloning of the QTL *Nidd/DBA* has identified four genes as potential T2D candidates as they display a differential expression in pancreatic islets and/or sequence variation. However, it cannot be excluded that genetic variants, including non-coding SNPs, indels, or copy number polymorphisms, may exert effects on regulatory circuits in the locus, thereby affecting islet cell function and glycemic control. Similarly, it must be considered that the *Nidd/DBA* locus may contain a diabetogenic variant not in a gene but rather in a regulatory region.

## Conclusion

In conclusion, the integration of comparative analysis of multiple inbred populations with one common breeding partner, haplotype mapping, expression, and sequence data substantially improved the mapping resolution of the diabetes QTL *Nidd/DBA*. Within the critical fragment of 3.3 Mbp, four genes with differential expression in pancreatic islets and/or genetic variants were identified. Future studies are necessary to validate the role of the different candidates in β-cell function and for maintaining glycemic control. This information may finally contribute to our growing understanding of the pathomechanisms that lead to β-cell failure and provide new paths toward targeted therapies and prevention strategies.

## Data Availability Statement

The data discussed in this publication have been deposited in NCBI’s Gene Expression Omnibus (Edgar et al., 2002) and are accessible through GEO Series accession number GSE152127 (https://www.ncbi.nlm.nih.gov/geo/query/acc.cgi?acc=GSE152127).

## Ethics Statement

The animal study was reviewed and approved by State Agency of Environment, Health, and Consumer Protection (State of Brandenburg, Germany).

## Author Contributions

HA designed and performed the experiments, wrote and edited the manuscript. NH designed and performed the experiments and interpreted the data. HA and NH equally contributed on the composition and the analyses conducted in the manuscript. PG and MJ analyzed the data. SO performed the experiments. GS analyzed the data. AK performed the experiments and analyzed the data. DA analyzed the data and provided expertise. GB provided expertise. TS and SL performed the experiments and provided the data. AC, HA-H, and H-GJ were involved in designing the study. AS designed and directed the study and edited the manuscript. HV designed and directed the study, wrote and edited the manuscript. All authors contributed to the article and approved the submitted version.

### Conflict of Interest

The authors declare that the research was conducted in the absence of any commercial or financial relationships that could be construed as a potential conflict of interest.

## Footnotes

^1^https://www.sanger.ac.uk/sanger/Mouse_SnpViewer/rel-1303

^2^https://www.mousephenotype.org/
