# Supplementary material for: Identification of Novel Potential Type 2 Diabetes Genes Mediating β-Cell Loss and Hyperglycemia Using Positional Cloning
Source: Front Genet. 2020 Sep 30;11:567191. doi: 10.3389/fgene.2020.567191 (PMC7561370; doi:10.3389/fgene.2020.567191)
Supplement: Supplementary file 1 [file Data_Sheet_1.docx]

**Supplementary Material**

**Supplementary Table 1. List of SNPs and microsatellite markers with primer sequences used for genotyping of congenic mice (GRCm38).**

| **Position (Mbp)** | **Identifier** |
| --- | --- |
| **82.81** | **rs3692661** |
| **103.94** | **rs32350037** |
| **105.29** | **D4Mit306** |
| **106.14** | **rs257363923** |
| **108.14** | **rs233757491** |
| **111.47** | **rs32727600** |
| **112.61** | **rs108436357** |
| **114.10** | **rs219850479** |
| **114.23** | **rs51626248** |
| **114.40** | **rs46052154** |
| **114.49** | **D4Mit332** |
| **117.15** | **rs247210886** |
| **117.45** | **D4Mit122** |
| **127.72** | **rs3674038** |

**Supplementary Table 2. Analysis of putative transcription factor binding sites (TFBS).**The binding affinity score of NZO and DBA reflect the predicted stability of a transcription (TF) binding to a TFBS. In turn, the absolute change of the binding affinity describes the difference of the TF-binding to the DBA sequence due to the indicated SNP.

**Figure Legends**

**Supplementary Figure S1. Representative images of pancreatic histology in *Nidd/DBA* mice.**The pancreas was excised from male *Nidd/DBA^N/N^*, *Nidd/DBA^N/D^*and *Nidd/DBA^D/D^* mice (RCS-I, *Nidd/DBA.13.6*) at different time points (n=3-8 animals/genotype). Tissue sections were stained with an antibody against insulin and scanned with a MIRAX MIDI scanner. Representative images of insulin-DAB staining of islets (brown), in addition to hematoxylin stained nucleic acids (purple) and eosin stained proteins (pink). Scale bar: 2000 µm (whole pancreas section); 500 µm (zoomed-in).
