# Supplementary figures and images for: Identification of Novel Potential Type 2 Diabetes Genes Mediating β-Cell Loss and Hyperglycemia Using Positional Cloning
Source: Front Genet. 2020 Sep 30;11:567191. doi: 10.3389/fgene.2020.567191 (PMC7561370; doi:10.3389/fgene.2020.567191)

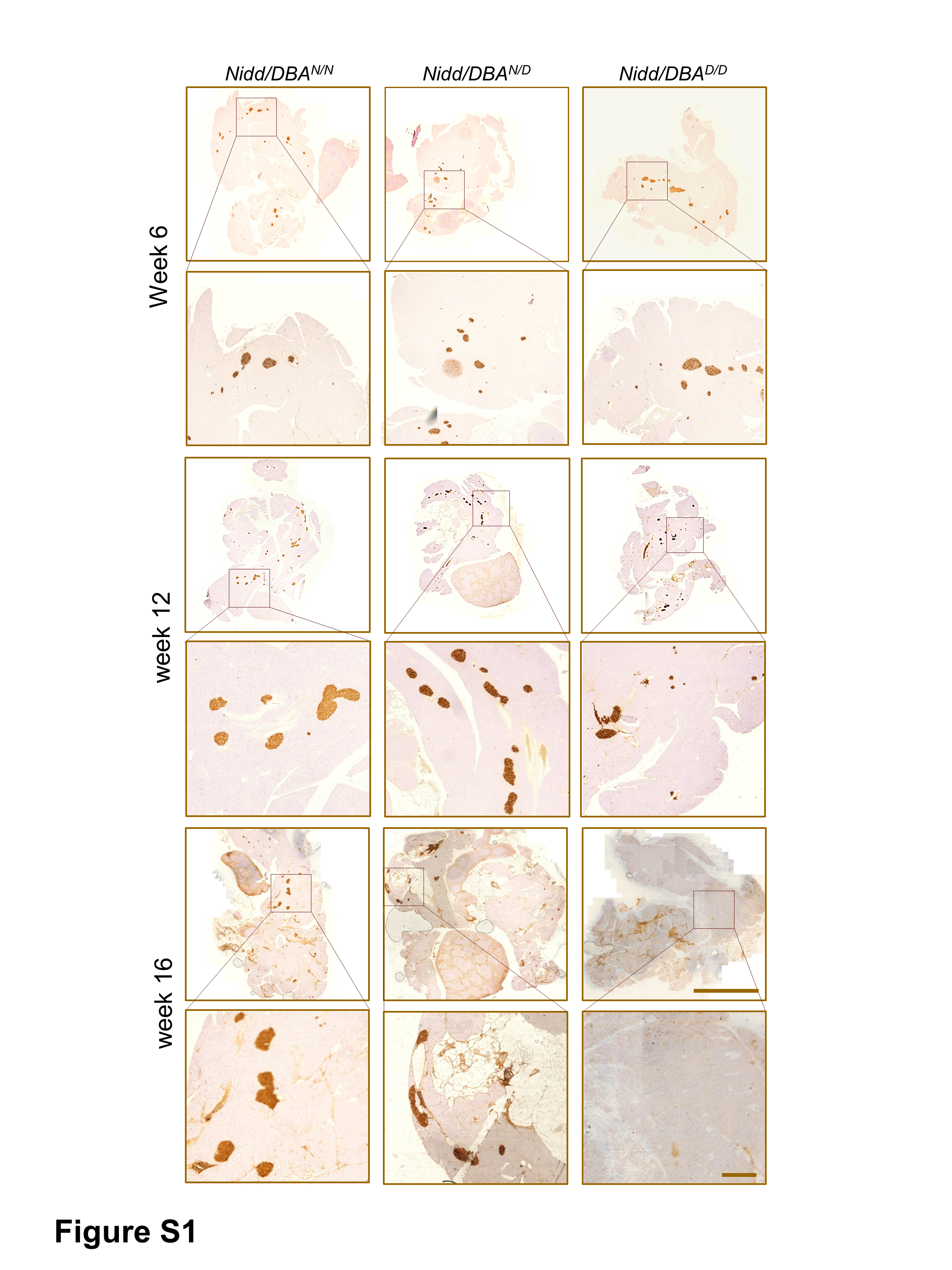

Supplement: Supplementary file 2 [file Image_1.tif]
